# Successful treatment of five cases of catatonia treated with guanfacine without ECT: a case series from a psychiatric hospital in Japan

**DOI:** 10.1186/s12888-025-07349-3

**Published:** 2025-10-01

**Authors:** Hiroki Saito, Mikael Tiger, Ryosuke Arakawa, Amane Tateno

**Affiliations:** 1 Onda-Daini Hospital, Matsudo-Shi, Chiba Japan; 2 Centre for Psychiatry Research, Department of Clinical Neuroscience, Karolinska Institutet & Stockholm Health Care Services, Stockholm County Council, Stockholm, Sweden; 3 Department of Pharmacology, Graduate School of Medicine, Nippon Medical School, Tokyo, Japan; 4 Department of Neuropsychiatry, Graduate School of Medicine, Nippon Medical School, Sendagi 1-1-5, Bunkyo-Ku, Tokyo, 113-8602 Japan

**Keywords:** Catatonia, Guanfacine, Schizophrenia, Schizoaffective disorder

## Abstract

**Background:**

Catatonia, including malignant catatonia, is a severe neuropsychiatric condition historically associated with schizophrenia or schizoaffective disorder. However, recent diagnostic frameworks, such as the Diagnostic and Statistical Manual of Mental Disorders, Fifth Edition, Text Revision (DSM-5-TR) and the International Classification of Diseases, 11th Revision (ICD-11), offer a broader understanding. These systems classify catatonia not as a standalone diagnosis but as a specifier that may occur alongside various psychiatric disorders, including schizophrenia spectrum disorders, mood disorders, neurodevelopmental disorders, or medical conditions. Although electroconvulsive therapy (ECT) and benzodiazepines are considered gold standard treatments for catatonia, many healthcare settings lack access to ECT, and certain physical conditions make ECT relatively contraindicated or a high-risk option. Guanfacine, a central adrenergic α2A receptor agonist, has a similar mechanism of action as the sedative dexmedetomidine, which has shown efficacy in treating catatonia. However, the effectiveness of guanfacine in this context has not been tested.

**Case presentation:**

We report five cases of catatonia (including malignant catatonia) associated with schizophrenia or schizoaffective disorder. These cases were treated either in a psychiatric hospital or an outpatient clinic, both of which lacked access to ECT. Extended-release formulation of guanfacine was administered alongside temporary benzodiazepine use, and treatment outcomes were observed. All five patients were in syndromal remission from catatonia following treatment that included guanfacine in combination with other pharmacological interventions. Mild to moderate side effects were observed. These included dizziness and fatigue in one patient, and hypotension and bradycardia in two others. All adverse effects resolved with dose reduction. Complications of catatonia included impaired oral intake requiring nutritional support in two patients and urinary catheterization due to immobility in two patients.

**Conclusions:**

These findings suggest that guanfacine may serve as a safe and effective alternative to ECT for catatonia in settings where ECT is unavailable or relatively contraindicated. The clinical courses also suggest that dysfunction of the central noradrenergic system may contribute to the pathophysiology of catatonia, and that guanfacine’s selective α2A adrenoceptor modulation may play a role in symptom improvement. Further research is needed to validate these findings and clarify the underlying mechanisms.

## Background

Catatonia is a complex neuropsychiatric disorder characterized by a wide range of motor signs (such as rigor, dyskinesia, negativism, posturing, catalepsy, gegenhalten and/or stereotypies), affective signs (including anxiety, flat affect, affective lability, aggression, impulsivity, and/or combativeness), and cognitive–behavioural signs (such as mutism, echolalia, echopraxia and verbigeration) [1]. While historically strongly associated with schizophrenia or schizoaffective disorder [2, 3], catatonia is now understood in a more nuanced way in terms of its presentation and etiology, as reflected in current diagnostic classifications, including DSM-5-TR [4] and ICD-11 [5]. Both systems classify catatonia not as a standalone disorder, but as a specifier that can accompany various mental disorders (such as schizophrenia spectrum disorders, mood disorders, or other mental disorders) or medical conditions. This broad classification acknowledges that its presence doesn't automatically imply a primary psychotic disorder, as it can arise from a wide range of psychiatric and medical etiologies. Malignant catatonia is a severe form of catatonia characterized by the coexistence of catatonic symptoms with autonomic abnormalities such as fever, unstable blood pressure, tachycardia, and diaphoresis, often accompanied by delirium and muscle rigidity [6]. If left untreated, this condition can be life-threatening [6–8]. While not a distinct diagnostic entity in the DSM-5-TR or ICD-11, its recognition underscores the critical need for a comprehensive assessment to determine the underlying etiology of catatonia, which has significant implications for treatment strategies. Until now, electroconvulsive therapy (ECT) in addition to benzodiazepines has been considered an effective treatment for catatonia [1, 9]. However, catatonia often occurs in settings where ECT is not a feasible treatment option, such as psychiatric hospitals or outpatient clinics without ECT facilities. Previous studies have reported that catatonia is frequently observed in acute psychiatric inpatients, with prevalence estimates approximately 10% [10]. Additionally, physical conditions such as relative contraindications [11] may preclude the use of ECT. Therefore, pharmacological alternatives to ECT are warranted.

Dexmedetomidine is a central adrenergic α2 receptor agonist that reduces noradrenaline activity in the central nervous system and inhibits the sympathetic nervous system [12, 13]. Although dexmedetomidine is a medication primarily used in an Intensive Care Unit (ICU) as a sedative-hypnotic agent [12], sublingual dexmedetomidine is also used and is effective for the treatment of agitation in patients with schizophrenia and bipolar disorder [14]. Moreover, recent case reports suggest that dexmedetomidine is also effective in the treatment of catatonia [15, 16].

Guanfacine is an anti- Attention-Deficit/Hyperactivity Disorder (ADHD) drug that is also approved for use in children [17]. Its mechanism of action is similar to dexmedetomidine, acting as a central adrenergic α2A receptor agonist [18–21], and it is widely used in psychiatric settings as an anti-ADHD drug, even in psychiatric hospitals or outpatient clinics without ECT or ICU facilities. However, whether guanfacine is effective and safe for the treatment of catatonia has not been determined.

Here, we report five cases of catatonia associated with schizophrenia or schizoaffective disorder (including malignant catatonia) in a psychiatric inpatient unit or an outpatient clinic where ECT was unavailable. The use of the extended-release formulation of guanfacine as an alternative to ECT, alongside temporary benzodiazepine treatment, resulted in effective and safe remission.

## Case series presentation

All patients in this case series were treated at Onda-daini Hospital, a psychiatric hospital without ECT capabilities. All patients were diagnosed with catatonia according to the DSM-5-TR criteria. Table 1 presents which specific diagnostic criteria were met before treatment and whether those symptoms resolved following treatment. In this case series, guanfacine refers to the extended-release formulation, which is the only type approved and available for clinical use in Japan.

**Table 1:** The ± notation indicates presence or absence of signs of catatonia before and after treatment

Meets DSM-5-TR Criteria/Criteria after treatment	Patient 1	Patient 2	Patient 3	Patient 4	Patient 5
1. Stupor	+/-	-/-	-/-	+/-	-/-
2. Catalepsy	+/-	-/-	-/-	+/-	-/-
3. Waxy flexibility	+/-	+/-	+/-	+/-	-/-
4. Mutism	+/-	+/-	+/-	+/-	+/-
5. Negativism	+/-	+/-	+/-	+/-	+/-
6. Posturing	+/-	-/-	-/-	+/-	-/-
7. Mannerism	-/-	-/-	-/-	-/-	+/-
8. Stereotypy	-/-	+/-	-/-	-/-	+/-
9. Agitation not influenced by external stimuli	-/-	-/-	-/-	+/-	+/-
10. Grimacing	+/-	+/-	+/-	+/-	+/-
11. Echolalia	-/-	-/-	-/-	-/-	+/-
12. Echopraxia	-/-	-/-	-/-	-/-	-/-

### Patient 1

**Fig. 1:**
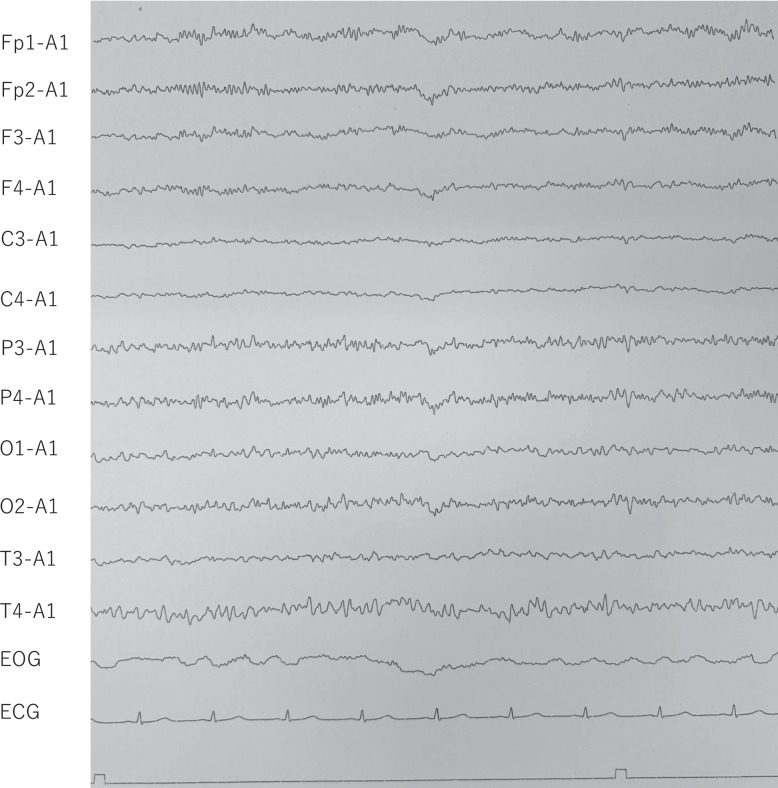
EEG (day10)

**Fig. 2:**
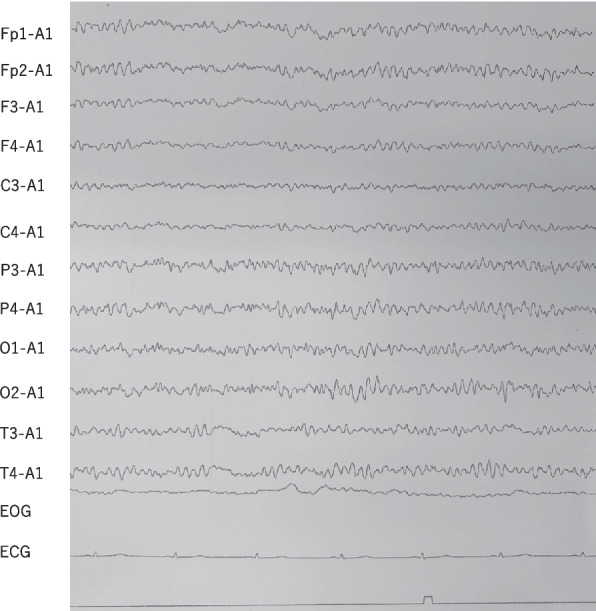
EEG (day45)

A 57-year-old woman with a prior diagnosis of schizoaffective disorder had been treated with olanzapine 5 mg and lorazepam 1 mg. After undergoing pylorus-preserving gastrectomy for a gastrointestinal stromal tumor in a general medical ward, she developed hallucinations and delusions, accompanied by psychomotor agitation, echolalia, and disorganized behavior such as urinating outside the toilet. Retrospectively, these were early signs of catatonia, although no psychiatrist was available at the previous hospital and she was managed by internal medicine staff. Initially, her presentation was considered to indicate an exacerbation of schizoaffective disorder combined with postoperative delirium. Within two weeks, lorazepam 1 mg was discontinued, olanzapine was increased from 5 to 20 mg, and due to persistent hallucinations and delusions, intravenous haloperidol 5 mg was administered as needed. She subsequently developed a body temperature (BT) of 40 °C, along with diaphoresis, muscular rigidity, and an elevated creatine kinase (CK) level of 6952 IU/L, which collectively raised suspicion for neuroleptic malignant syndrome (NMS) [22]. All antipsychotics were discontinued, and the management included intravenous fluids at 3000 mL/day, intravenous dantrolene 20–40 mg, and oral lorazepam 1.5 mg. Her CK briefly peaked at 18,566 IU/L, but declined to 1004 IU/L over ten days following dantrolene treatment. Despite this biochemical improvement, her catatonic features, including stupor and muscular rigidity, as well as autonomic abnormalities such as persistent high fever and diaphoresis, showed no improvement. Symptomatic control of the fever was attempted using naproxen, but this approach was ineffective. Since management in the general medicine ward was deemed inadequate without psychiatric oversight, and although her biochemical markers had started to improve, she was transferred to our hospital. Upon admission, her clinical presentation was reassessed, leading to a re-diagnosis of malignant catatonia. Her vital signs at the admission indicated significant autonomic abnormalities, with a blood pressure (BP) of 175/110 mmHg, a heart rate (HR) of 127 bpm, and a BT of 38.4 °C. In addition, she exhibited profuse sweating, further indicating autonomic dysregulation. Concurrently, she exhibited clear catatonic signs, including catalepsy, muscular rigidity, mutism, negativism, grimacing, and stupor, fulfilling the diagnostic criteria for catatonia according to DSM-5-TR. These findings, including autonomic abnormalities and catatonia, collectively indicated persistent malignant catatonia. Whole-body computed tomography (CT), including the head, revealed no significant abnormalities. Furthermore, blood tests, including assessments of thyroid and adrenal function, excluded the possibility of acute endocrine crisis states or infections. Meanwhile, the CK level was still elevated at 1845 U/L. Due to difficulty with oral administration of medications and nutrition, intravenous fluid therapy (2000 mL/day) and a urinary catheter were required. On Day 1, we promptly administered an intravenous injection of 6 mg of lorazepam, which is the first-line treatment for malignant catatonia. After one week (at Day 7), her symptoms had partially improved; however, stupor and akinesia persisted, and she remained unable to receive oral nutrition, necessitating continuous intravenous infusion. Her vital signs were still indicative of autonomic dysregulation, with a systolic blood pressure of 150–160 mmHg, BT of 37–38 °C, and HR of 110–120 bpm. Therefore, we considered transferring the patient to another hospital for the purpose of ECT, as our hospital does not have the facilities to perform ECT. However, no other hospitals were able to promptly accept her transfer. While awaiting transfer for ECT, we considered administering guanfacine, which constitutes off-label use, with the consent of the patient's family. On Day 7, we initiated an oral dose of guanfacine at 2 mg. By the evening of the same day, she was able to voluntarily open and close her hands and extend and flex both lower limbs in response to instructions. On Day 8, blood tests revealed that CK levels had peaked out at 172 U/L. Although she remained dominated by hallucinations and delusions, making her speech incoherent, she was able to engage in conversation, and sustained compliance with instructed movements was observed. Additionally, she was able to ingest one bottle of oral liquid nutrition. By Day 10, her vital signs stabilized (BP 116/81 mmHg, HR 86 bpm, BT 36.6 °C), and recovery from catatonia became evident. Electroencephalography (EEG) showed predominantly fast waves, likely due to benzodiazepine use (Fig. 1), but remained within normal limits, ruling out consciousness disturbances, delirium, and epilepsy. At this stage, she was able to transfer to a wheelchair and began rehabilitation. On Day 14, although she was able to engage in conversation, hallucinations and delusions persisted significantly. Olanzapine 2.5 mg was initiated for the treatment of schizoaffective disorder. Since intravenous lorazepam 6 mg was still required, guanfacine was subsequently increased to 3 mg to maintain autonomic stability and support the tapering of high-dose lorazepam, which was gradually discontinued due to concerns about prolonged use, such as oversedation, delirium, and increased risk of falls. Between Day 15 and Day 17, lorazepam was transitioned from intravenous infusion (6 mg) to oral administration (3 mg). Oral intake of liquid nutrition increased to three bottles per day, while intravenous fluids were reduced from 2000 mL/day to 1000 mL/day. The speech influenced by hallucinations and delusions gradually improved with olanzapine 2.5 mg. On Day 17, the diet was advanced from oral liquid nutrition to a minced diet, and intravenous fluids were discontinued. As independent urination and defecation were confirmed, the urinary catheter was removed. However, disorganized speech was still observed at night, so olanzapine was increased to 5 mg. On Day 18, the communication further improved, and she was able to stand and take a shower. On Day 21, persecutory delusions such as “being watched” and “being targeted” persisted, prompting an increase in olanzapine to 7.5 mg. Guanfacine was further increased to 4 mg to enhance autonomic regulation during the up-titration of olanzapine and continued tapering of lorazepam, aiming to minimize the risk of catatonia recurrence while avoiding the adverse effects associated with long-term benzodiazepine use. On Day 24, mild hypotension (BP 85/54 mmHg, HR 69 bpm, BT 36.6 °C) and dizziness were observed. Thus, lorazepam was reduced from 3 to 2 mg. Around this time, she engaged more in daily activities, such as chatting with other patients and watching television in the dayroom. On Day 28, she was able to walk within the hospital accompanied by staff, and by Day 31, as hypotension (BP 85/62 mmHg, HR 62 bpm, BT 36.6 °C) and dizziness persisted, lorazepam was further reduced to 1.5 mg. On Day 35, considering the continued hypotension (BP 86/56 mmHg, HR 75 bpm, BT 36.6 °C) and dizziness, lorazepam was reduced to 1 mg, and independent outings from the hospital were permitted. On Day 41, lorazepam was tapered to 0.5 mg, and by Day 42, it was completely discontinued. During this period, she continued to spend time calmly, engaging in outings, watching television in the dayroom, and conversing with other patients. There was no recurrence of catatonia, and her hallucinations and delusions improved. Her EEG on Day 45 showed improvement to normal patterns (Fig. 2). On Day 50, mild insomnia was reported, so quetiapine 25 mg was added, leading to improvement in sleep disturbances. Finally, on Day 54, she was discharged to her home with stable vital signs (BP 119/94 mmHg, HR 77 bpm, BT 36.3 °C). She maintained remission with guanfacine 4 mg, olanzapine 7.5 mg, and quetiapine 25 mg, without requiring further benzodiazepines. Since discharge, she has been living a normal daily life with her family.

### Patient 2

A 54-year-old woman with no prior history of psychiatric illness was admitted to our hospital with a diagnosis of schizophrenia. She was found to have hallucinations and delusions, including statements such as “I am being spoken ill of,” “I am being targeted,” and “I will be killed.” Whole-body CT, including the head, revealed no significant abnormalities, and her EEG was normal, ruling out disturbances of consciousness and delirium and epilepsy. Furthermore, blood tests, including assessments of thyroid and adrenal function, excluded the possibility of acute endocrine crisis states or infections. Pharmacological treatment was initiated with oral risperidone at 4 mg and olanzapine at 10 mg, which were administered simultaneously to address the acute psychotic symptoms. As her hallucinations and delusions gradually subsided and clinical remission was achieved, risperidone was discontinued and replaced with paliperidone long-acting injection (LAI) at 150 mg every four weeks. This transition was made to ensure long-term adherence. Olanzapine at 10 mg was temporarily continued to maintain stability during the switch. Following the standard initiation protocol, a 100 mg dose of paliperidone LAI was administered one week after the 150 mg injection. No adverse effects or complications were observed, and the patient remained in remission. After a one-week observation period post-injection of the paliperidone LAI 100 mg, she was discharged to her home. At a scheduled outpatient follow-up three weeks after discharge, the second dose of paliperidone LAI at 150 mg was administered, and olanzapine was discontinued to simplify the regimen and achieve antipsychotic monotherapy. Approximately two weeks later, during a follow-up visit to our hospital, she only whispered and habitually stated that, “My body movements have become like a robot’s.” She presented with a grimacing expression, muscle rigidity, and mutism, meeting the diagnostic criteria for catatonia as defined in the DSM-5-TR. In the outpatient setting, she was diagnosed with catatonia and treated with oral lorazepam at 3 mg, but her symptoms only partially improved. Paliperidone LAI 150 mg/4 weeks was discontinued. Muscle rigidity was subsequently determined to be an extrapyramidal symptom, and biperiden at 3 mg was administered as an antiparkinsonian agent. However, it was ineffective. As she became unable to consume solid food and required oral liquid nutritional supplements, we initiated guanfacine at 2 mg to enhance her pharmacological treatment. Over the course of a few weeks, her symptoms improved dramatically; her muscle rigidity and grimacing resolved, and she regained the ability to move and eat solid food. Biperiden and lorazepam were gradually tapered off and discontinued. Given that remission was achieved after the initiation of guanfacine, it is likely that this agent played an important role in the patient’s recovery. However, it is also possible that the discontinuation of paliperidone LAI contributed to sustained improvement by removing a potential exacerbating factor. For the original pathological condition of schizophrenia, we initiated olanzapine at 2.5 mg, which was subsequently reduced to 1.25 mg in response to complaints of drowsiness. As complaints of dizziness and fatigue persisted, guanfacine was reduced from 2 to 1 mg, which led to an improvement in these symptoms. In the end, remission was maintained with guanfacine at 1 mg and olanzapine at 1.25 mg. Since then, she has been living a normal daily life at home with her family.

### Patient 3

A 56-year-old man with a history of schizophrenia and two prior hospitalizations for hallucinations and delusional states had been well stabilized on paliperidone LAI at 150 mg every four weeks. While he was hospitalized in a general medical ward due to a COVID-19 infection, he received the scheduled paliperidone LAI 150 mg injection without any immediate adverse reactions, consistent with his usual regimen. However, several days after this administration and shortly after recovering from COVID-19, he developed mutism and muscular rigidity in both upper and lower limbs, accompanied by significant impairment of motor activity. Due to severe motor impairment, he was unable to get up from bed and required a urinary catheter. Initially, parkinsonism was suspected, and trihexyphenidyl hydrochloride 6 mg and ethyl loflazepate 2 mg were administered; however, there was no improvement. He was then transferred to our hospital. At the admission, he presented with a grimacing expression, muscle rigidity, and mutism. These symptoms met the diagnostic criteria for catatonia as defined in the DSM-5-TR. Head CT and chest radiography revealed no significant abnormalities. Electrocardiography also showed no abnormalities. Furthermore, blood tests, including assessments of thyroid and adrenal function, excluded the possibility of acute endocrine crisis states or infections. Given his recent COVID-19 infection and the associated risk of respiratory complications, we chose to initiate guanfacine at 2 mg rather than increasing benzodiazepines. Guanfacine treatment was associated with improved movement in all limbs, resolution of mutism, and removal of the urinary catheter within a few days. The guanfacine dose was subsequently increased to 3 mg to support further improvement. At the same time, due to concerns that the catatonia may have been exacerbated by decreased medication tolerability in the context of his recent infection, the paliperidone LAI dose was reduced to 100 mg at the time of his next scheduled injection. The doses of trihexyphenidyl and ethyl loflazepate were gradually tapered off and discontinued. Due to mild hypotension and bradycardia, with a BP of approximately 90/60 mmHg and an HR of around 50 bpm, the dose of guanfacine was tapered down to 1 mg. Subsequently, vital signs returned to normal, with a BP of 103/74 mmHg and an HR of 74 bpm, and he remained in remission from psychiatric symptoms. Then he was discharged to his home.

### Patient 4

A 62-year-old man with a history of schizophrenia has been hospitalized three times due to hallucinatory and delusional episodes. He has been maintained on risperidone 7 mg and levomepromazine 10 mg. He was admitted to our hospital due to worsening delusional persecution directed toward his cohabiting father. Upon admission, he exhibited an explosive state of agitation. A comprehensive laboratory evaluation was performed at the time of admission, including assessments of thyroid function, adrenal function, electrolytes, and markers of infection or inflammation. All values were within normal limits, and no signs of an underlying organic etiology for catatonia were found. Treatment with risperidone (up to 7 mg) and intramuscular injections of olanzapine 10 mg was attempted but remained ineffective. Following these pharmacological interventions, he gradually developed symptoms suggestive of mania, including pressured speech, flight of ideas, and grandiose ideation. Based on these findings, he was clinically assessed as exhibiting manic agitation, and lithium carbonate 400 mg was initiated. Subsequently, he developed stupor, muscular rigidity, and mutism. He was diagnosed with catatonia, and given the close temporal association between lithium administration and the onset of catatonia, lithium carbonate was discontinued based on the clinical judgment that it may have contributed to the condition. Intravenous administration of lorazepam 2 mg and oral administration of lorazepam 3 mg were started. The intravenous formulation was discontinued due to repeated self-removal of the intravenous lines by the patient. Oral lorazepam was administered at 3 mg daily, the maximum recommended dose in Japan, and was not increased further. While the stupor was resolved, explosive agitation without external stimulus persisted. As an enhancement to pharmacological therapy, guanfacine 2 mg was initiated and titrated up to 6 mg. Over time, agitation and hallucinatory-delusional states improved, and lorazepam was no longer required. At the same time, risperidone 7 mg, which was identified as the causative agent of catatonia in this case, was gradually tapered off and replaced with brexpiprazole 2 mg. Subsequently, mild hypotension and bradycardia were observed, with a BP in the range of 90/60 mmHg and an HR in the range of 50 bpm. As a result, the dose of guanfacine was gradually tapered down to 4 mg. Ultimately, remission was maintained with brexpiprazole at 2 mg and guanfacine at 4 mg, and vital signs returned to normal, with a BP of 119/79 mmHg and an HR of 62 bpm. After this, he was discharged to his home.

### Patient 5

A 63-year-old man with a history of multiple hospitalizations for schizoaffective disorder was admitted due to a depressive state. At the time of admission, he exhibited depressive symptoms such as decreased motivation and fatigue. He had been maintained on risperidone LAI 25 mg/2 weeks, levomepromazine 50 mg, valproate 400 mg, carbamazepine 600 mg, sertraline 50 mg, etizolam 3 mg, nitrazepam 5 mg, and triazolam 0.25 mg. He had been prescribed long-term benzodiazepine therapy, including etizolam, nitrazepam, and triazolam, for several years prior to admission. Due to repeated episodes of falling, including one that resulted in major bleeding from a temporal artery injury, it became clinically necessary to taper and discontinue these medications. Subsequently, he presented with mutism, negativism, mannerisms, stereotypy, agitation not influenced by external stimuli, grimacing, and echolalia, leading to a diagnosis of catatonia. Given the close temporal relationship between benzodiazepine discontinuation and the onset of symptoms, benzodiazepine withdrawal was considered a likely contributing factor. Risperidone LAI, levomepromazine, and sertraline were discontinued. The decision to discontinue sertraline was based on concerns that its serotonergic activity might exacerbate agitation, which was one of the patient’s prominent symptoms at the time, and because he was no longer exhibiting depressive symptoms. The primary medication was subsequently switched to brexpiprazole 1 mg. However, the symptoms persisted. Intramuscular administration of diazepam 5 mg alleviated akinesia, negativism, and agitation to some extent, enabling the patient to take medications orally on a regular basis. Oral administration of guanfacine 2 mg and lorazepam 3 mg was initiated, leading to gradual symptom improvement. Because of postural instability, lorazepam was gradually reduced to 0.5 mg, while guanfacine was simultaneously increased to 3 mg. Due to findings suggesting hyponatremia (serum sodium 128 mEq/L) and drug-induced syndrome of inappropriate secretion of antidiuretic hormone, carbamazepine was replaced with quetiapine 100 mg. Finally, remission was maintained with brexpiprazole 2 mg, quetiapine 100 mg, guanfacine 3 mg, and lorazepam 0.5 mg, and he was discharged to his home.

## Discussion

In the present five cases, we observed that treatment courses involving guanfacine were associated with clinical improvement in catatonia, including malignant catatonia. However, it is important to note that these improvements may also have resulted from other pharmacological interventions, such as benzodiazepine administration or adjustments of antipsychotic medications, spontaneous remission, or the natural course of the illness. Guanfacine was selected because of its pharmacological similarity to dexmedetomidine, an α2 adrenergic agonist that has shown potential effectiveness in treating catatonia in recent case reports [15, 16]. Since sublingual dexmedetomidine is not approved for clinical use in Japan, we administered oral guanfacine as an alternative. Furthermore, previous studies in animal models have reported that clonidine, which shares a similar mechanism of action with both guanfacine and dexmedetomidine, counteracted perphenazine-induced catatonia and demonstrated anti-catatonic effects [23, 24]. Therefore, while guanfacine may have contributed to the clinical outcomes observed, this remains a hypothesis-generating observation that requires confirmation through controlled studies. During treatment, no serious adverse events occurred. However, mild to moderate adverse effects such as dizziness, fatigue, hypotension, and bradycardia were observed in several patients. These side effects were effectively managed through dose reduction of guanfacine, allowing treatment to continue safely. Catatonia often occurs in psychiatric hospitals or outpatient clinics without access to ECT [9]. Moreover, it can occur in patients for whom ECT is relatively contraindicated or poses a high risk due to physical conditions [11, 25]. Therefore, the use of guanfacine as an alternative to ECT for treating catatonia could be a potential option for patients unable to undergo ECT. However, the present study does not constitute a randomized controlled trial (RCT), which is considered the gold standard for evaluating the efficacy and safety of treatments. Conducting an RCT that directly compares an ECT group with a guanfacine group would be crucial to provide robust and reliable evidence of the effect of guanfacine. If the non-inferiority of guanfacine was demonstrated, it would strengthen our findings. However, the lack of randomization in this study necessitates caution in interpreting the results, emphasizing the need for future RCTs and rigorous research.

Here, we note a clinically important observation from our experiences. In this case series, complex pharmacological regimens appeared to be contributing factors in the development or exacerbation of catatonia. Notably, in Patient 1, catatonic symptoms emerged following the combined use of olanzapine 20 mg and haloperidol 5 mg. In Patient 2, catatonia developed after the introduction of paliperidone LAI 150 mg while olanzapine 10 mg was concurrently administered. Similarly, in Patient 3, catatonia appeared after paliperidone LAI 150 mg administration. In Patient 5, abrupt discontinuation of benzodiazepines was associated with the onset of catatonia. These observations suggest that both antipsychotic polypharmacy and sudden benzodiazepine withdrawal may play a role in triggering catatonic episodes. They highlight the importance of cautious dose titration, careful sequencing during medication transitions, and close clinical monitoring in the management of patients at risk for catatonia. Additionally, it is worth considering that in some cases within this series, the resolution of catatonia may have been partially influenced by dose reduction or discontinuation of second-generation antipsychotics. In Patient 2, catatonia remitted following the withdrawal of paliperidone LAI. Similarly, in Patient 3, a reduction in the dose of paliperidone LAI from 150 to 100 mg occurred prior to clinical improvement. Although these pharmacological adjustments were made alongside the introduction of guanfacine, it remains plausible that they contributed to the amelioration of catatonic symptoms. These observations underscore the importance of carefully evaluating the role of antipsychotic exposure in both the emergence and resolution of catatonia, especially in complex treatment regimens.

Regarding Patient 1, she initially presented at the previous hospital with a condition that is often similar to or co-occurs with malignant catatonia, specifically NMS [26, 27]. Dantrolene, the first-line treatment for NMS [22], was administered. It effectively reduced CK levels. However, catatonic symptoms and autonomic dysfunction did not improve. After the patient was admitted to our hospital, a revised diagnosis of malignant catatonia was established. Subsequent treatment in accordance with this diagnosis resulted in remission. In retrospect, the clinical course suggests a complex pathological condition involving both NMS and malignant catatonia. These syndromes exhibit significant overlap in symptoms, such as autonomic instability and muscular rigidity, and there is a growing body of literature proposing that they are considered to exist on the same spectrum of illness [26–30]. It is particularly noteworthy that α2 adrenergic receptor agonists, including clonidine and dexmedetomidine, have been reported to be effective in treating NMS [31–33]. For example, a case report demonstrated that the addition of clonidine to standard therapies such as dantrolene and benzodiazepines significantly reduced mortality in patients with NMS [31]. Based on this shared pharmacological profile, it is reasonable to interpret that guanfacine may have been effective in treating symptoms that were initially suspected to reflect NMS but later diagnosed and managed as malignant catatonia in this patient. Furthermore, both NMS and malignant catatonia frequently co-occur with delirium [6, 22, 34, 35], either as a comorbid condition or as an early symptom. In this case, postoperative delirium was initially suspected at the previous hospital, which led to the discontinuation of benzodiazepines and the administration of antipsychotics. At our facility, other potential medical conditions were carefully ruled out through imaging and laboratory tests. EEG conducted on Day 10 confirmed the absence of delirium and other consciousness disturbances. While we were able to rule out delirium in this specific patient, it's important to acknowledge that delirium frequently complicates catatonia in a general clinical context [35]. This co-occurrence presents a significant diagnostic and therapeutic challenge. Notably, there are several reports indicating the efficacy of alpha 2 A adrenergic receptor agonists such as clonidine, dexmedetomidine, and guanfacine in the treatment of delirium [36–39]. Recently, the American Psychiatric Association released comments regarding the management of catatonia in the consultation-liaison setting [40], particularly concerning cases complicated by delirium. This perspective is important not only for psychiatric hospitals and clinics but also for consultation-liaison services in general hospitals, where delirium is frequently encountered. Following this line of reasoning, guanfacine may contribute to the early therapeutic management of catatonia complicated by delirium and improve patient outcomes across diverse clinical settings.

In addition to the five successfully treated cases reported in this case series, we encountered another noteworthy case. A 53-year-old woman with treatment-resistant schizophrenia presented with persistent catatonia. She had been receiving long-term treatment with risperidone 12 mg, quetiapine 400 mg, and lorazepam 3 mg, yet remained in a prolonged state of stupor, negativism, and mutism, requiring nutritional support via a nasogastric tube. Upon the addition of guanfacine at 2 mg, she became able to engage in verbal communication, suggesting an improvement in her catatonic state. However, she reported,"This medication makes my mind feel scrambled. I don’t want to take it,"after which her hallucinations and delusions became pronounced, and guanfacine was discontinued. Eventually, clozapine was introduced, and she achieved remission with a daily dose of 200 mg. While this was a dropout case for guanfacine, discontinued at the patient’s request and from which no definitive conclusions can be drawn, we believe it's a valuable clinical experience worth documenting.

On the other hand, guanfacine, a medication that selectively suppresses the noradrenergic system, implicates through the clinical course of these patients that hyperactivity of the noradrenergic system may play a role in the pathophysiology of catatonia, including malignant catatonia. Several past studies have discussed the relationship between catatonia and the noradrenergic system. Research in rodents [24] has demonstrated that clonidine, an α2A adrenergic agonist, alleviates perphenazine-induced catatonia, and it has been suggested that this anticatatonic effect of clonidine may be attributed to its presynaptic action. Furthermore, as mentioned above, two case reports [15, 16] have documented the improvement of catatonia with dexmedetomidine, another α2A adrenergic agonist, further supporting the potential involvement of the noradrenergic system in catatonia. From a pharmacological perspective, the α2A adrenergic receptor in the locus coeruleus is located in both presynaptic and postsynaptic regions [41]. Clonidine has very potent presynaptic actions, reducing noradrenaline release and inhibiting the firing of noradrenergic neurons of the locus coeruleus [21]. The sedative effects of dexmedetomidine are also considered to be mediated via α2A adrenergic receptors in the locus coeruleus [42]. Regarding guanfacine, the inhibition of the noradrenergic system via the locus coeruleus has also been documented in previous studies [19, 20]. Additionally, studies conducted in primates have provided several important insights into the relationship between the noradrenergic system and catatonia. One study [43] demonstrated that guanfacine-induced postsynaptic stimulation of adrenergic α2A receptors enhances learning from errors and facilitates the flexible switching of complex cognitive states while updating attention sets and processing prediction errors in neurons within the dorsolateral prefrontal cortex, anterior cingulate cortex, and striatum. Another study [44] found that guanfacine-induced stimulation of adrenergic α2A receptors in the dorsolateral prefrontal cortex improved spatial working memory. Additionally, previous research has shown that this stimulation strengthens working memory networks by inhibiting cyclic adenosine monophosphate (cAMP)-hyperpolarization-activated cyclic nucleotide-gated (HCN) channel signaling in the prefrontal cortex [45]. Moreover, case reports [46, 47] suggest that transcranial direct-current stimulation (tDCS) targeting the dorsolateral prefrontal cortex contributes to improving symptoms in catatonia patients. Neuroimaging studies using functional magnetic resonance imaging (fMRI) [48] have also implicated abnormalities in the dorsolateral prefrontal cortex and anterior cingulate cortex as underlying factors in catatonia. Additionally, previous studies have suggested that guanfacine, which is often used off-label for various conditions beyond ADHD, such as schizotypal cognitive deficits, may exert its therapeutic effects by stimulating postsynaptic adrenergic α2A receptors specifically in the prefrontal cortex. This mechanism of action is thought to contribute to its potential benefits in treating psychiatric disorders, including schizophrenia spectrum disorders [49]. Viewed as a whole, these findings suggest that guanfacine-induced pre- and postsynaptic stimulation of adrenergic α2A receptors, particularly in regions such as the dorsolateral prefrontal cortex, anterior cingulate cortex, and locus coeruleus, may have contributed to the observed improvement in catatonia patients reported in these cases.

This study demonstrated that guanfacine was effective and can be used safely for the treatment of catatonia, including malignant catatonia. However, this finding is based on the clinical courses of only five cases. In the future, it will be necessary to accumulate additional cases of catatonia treated with guanfacine to further elucidate in which types of condition it can be both effective and safely administered.

Our study had several limitations. The primary limitation of this study is that it is not a randomized controlled trial. Ideally, a randomized controlled trial comparing an ECT group and a guanfacine group should be conducted. If the non-inferiority of the guanfacine group in treatment outcomes could be confirmed, the findings would be more reliable. Therefore, further accumulation of cases is necessary to corroborate guanfacine as a treatment option for catatonia. Secondly, this report is based on the observation of clinical courses and lacks confirmation of the underlying pathophysiology at the molecular level. While substantial evidence regarding brain imaging in catatonia patients has been accumulating [48], the utilization of molecular imaging modalities, such as Positron Emission Tomography, would be preferable to test the hypothesis of dysfunction of the central noradrenergic system prior to treatment and its normalization post-treatment. Such an approach would enable a more rigorous and precise assessment of the pathophysiology of catatonia and its therapeutic potential.

## Conclusion

Our findings suggest that guanfacine may represent a promising therapeutic option for catatonia, particularly when it is incorporated into a comprehensive treatment strategy. In all five cases, catatonic symptoms improved following the initiation of guanfacine. Although other factors such as the discontinuation or adjustment of antipsychotic medications may have contributed to clinical improvement, the consistent temporal association between the administration of guanfacine and the resolution of symptoms across cases supports its potential utility. Mild to moderate adverse effects were observed, including dizziness, fatigue, hypotension, and bradycardia. However, these were successfully managed through dose adjustments, and no serious adverse events occurred. These observations highlight the importance of individualized dosing and careful monitoring when using guanfacine in the treatment of catatonia.

While this finding is encouraging, we emphasize that the present study is descriptive in nature, and causality cannot be inferred. The results suggest a possible role of noradrenergic dysfunction in the pathophysiology of catatonia and highlight the need for further controlled investigations into guanfacine’s potential utility in this context.

## Data Availability

No datasets were generated or analysed during the current study.

## Abbreviations

DSM-5-TR: Diagnostic and Statistical Manual of Mental Disorders Fifth Edition, Text Revision
ICD-11: International Classification of Diseases, 11th Revision
ECT: Electroconvulsive therapy
ICU: Intensive Care Unit
ADHD: Attention-Deficit/Hyperactivity Disorder
BT: Body temperature
CK: Creatine kinase
NMS: Neuroleptic malignant syndrome
BP: Blood pressure
HR: Heart rate
CT: Computed tomography
EEG: Electroencephalography
LAI: Long-acting injection
RCT: Randomized controlled trial
cAMP: Cyclic adenosine monophosphate
HCN: Hyperpolarization-activated cyclic nucleotide-gated
tDCS: Transcranial direct-current stimulation
fMRI: Functional magnetic resonance imaging
